# 

**Published:** 1917-08-18

**Authors:** 


### CONTENTS.

PAGE

**Sir Alfred Keogh and the R.A.M.C.** ... ... 387

**The Exhaustion of the Medical Profession** ... 388

**Hospital and Institutional News** ... ... ... 389
Sir William Watson Cheyne, M.P.—The General Hospital, Birmingham, and the Plenum System—The Existing Evils and their Remedy—Bone-Setters and the Wounded—Medical Honours and Promotions—Some of the Recipients—A Proposed New Ambulance Corps—The Protection of Hospital Ships—The Limits to Educational Convalescence—Soldiers at School—An Over-subscribed Special Appeal—The Size of Convalescent Homes—The Leicester Home for Shell-Shocked Soldiers—Manchester Council for Venereal Disease—This Week's Drug Market—To Our Readers.

**The Massacre of the Innocents** ... ... ... 393

No State Licence Given to Murder.

**The Guardian of London's Health** ... ... 394
The Work of the Metropolitan Asylums Board.

**The Worst War of the World** ... ... ... 395
III. The United States Troops Visit London.

**Warrior Patients in the Voluntary Hospitals** 396
A Plea for Raising War Office Payments with a Flat Rate.

PAGE

**Physical Orthopædics...** ... ... ... ... 397

II. A Grave National Deficiency.

**Hospital Sunday in London** ... ... ... 399

Some History and its Teachings.

**The Editor's Letter-Box** ... ... ... ... 400
Nursing Affairs in Ireland.
What the Fighting Man Thinks of War.

**The Matrons' and Sisters' Department** ... 401
Matrons in Council: III. From an Old Shoe.
Round the Hospitals.
Miss L. V. Haughton better—Miss McCarthy's Promotion—Splendid Unity at Dewsbury—Will the Central Committee Exhibit Wisdom? Registration by Agreement still Open—When A Matron is Indispensable.

**Nature Studies in Macedonia** ... ... ... 404

**King Edward VII.'s Hospital, Cardiff** ... ... 406
Colonel Bruce Vaughan has a Good Time.

**The Institutional Worker** ... ... (Suppl.) 1
The Control of the Laundry: Answer to July Question.
Question for August.
Enquiries and Answers.
The Sick and in Need.
Miscellaneous.
Employment and Training.

